# Vertebral Body Stapling versus Bracing for Patients with High-Risk Moderate Idiopathic Scoliosis

**DOI:** 10.1155/2015/438452

**Published:** 2015-11-04

**Authors:** Laury Cuddihy, Aina J. Danielsson, Patrick J. Cahill, Amer F. Samdani, Harsh Grewal, John M. Richmond, M. J. Mulcahey, John P. Gaughan, M. Darryl Antonacci, Randal R. Betz

**Affiliations:** ^1^Institute for Spine & Scoliosis, 3100 Princeton Pike, Lawrenceville, NJ 08648, USA; ^2^Sahlgren University Hospital, Gothenburg University, 413 45 Gothenburg, Sweden; ^3^Children's Hospital of Philadelphia, 34th Street and Civic Center Boulevard, Philadelphia, PA 19104, USA; ^4^Shriners Hospitals for Children-Philadelphia, 3551 N Broad Street, Philadelphia, PA 19140, USA; ^5^Cooper Medical School at Rowan University, 3 Cooper Plaza, Suite 411, Camden, NJ 08103, USA; ^6^Tri Rivers Surgical Associates, 9104 Babcock Boulevard, Suite 2120, Pittsburgh, PA 15237, USA; ^7^Thomas Jefferson University, 901 Walnut Street, 6th Floor, Philadelphia, PA 19107, USA; ^8^Temple University School of Medicine, 3420 N Broad Street, Philadelphia, PA 19104, USA

## Abstract

*Purpose*. We report a comparison study of vertebral body stapling (VBS) versus a matched bracing cohort for immature patients with moderate (25 to 44°) idiopathic scoliosis (IS). *Methods*. 42 of 49 consecutive patients (86%) with IS were treated with VBS and followed for a minimum of 2 years. They were compared to 121 braced patients meeting identical inclusion criteria. 52 patients (66 curves) were matched according to age at start of treatment (10.6 years versus 11.1 years, resp. [*P* = 0.07]) and gender. *Results*. For thoracic curves 25–34°, VBS had a success rate (defined as curve progression <10°) of 81% versus 61% for bracing (*P* = 0.16). In thoracic curves 35–44°, VBS and bracing both had a poor success rate. For lumbar curves, success rates were similar in both groups for curves measuring 25–34°. *Conclusion*. In this comparison of two cohorts of patients with high-risk (Risser 0-1) moderate IS (25–44°), in smaller thoracic curves (25–34°) VBS provided better results as a clinical trend as compared to bracing. VBS was found not to be effective for thoracic curves ≥35°. For lumbar curves measuring 25–34°, results appear to be similar for both VBS and bracing, at 80% success.

## 1. Introduction

The available nonsurgical treatment methods for moderate juvenile and adolescent idiopathic scoliosis (IS) are observation and bracing. Bracing has been the subject of great debate in recent years. The previously performed Scoliosis Research Society (SRS) brace study from the early 1980s attempted to prove effectiveness of conservative treatment of adolescent IS (AIS) [1]. A more recent follow-up of some of these patients showed that a meticulously performed brace regimen did hinder progression, with no need for subsequent surgical correction [2]. However, Dolan and Weinstein [3] conducted a systematic review of the available clinical studies for bracing and observation and compared surgical rates after observation and bracing for AIS. They concluded that there was not enough evidence to recommend bracing over observation, as the pooled surgical rate for bracing was 23%, and for observation it was 22%. Following this review, a randomized trial addressing the effect of bracing was conducted [4]. They were able to show that bracing is effective in decreasing the progression of high-risk [5] curves to the threshold for surgery.

There are other issues than the effectiveness associated with bracing, particularly the psychosocial ramifications of brace wear. Compliance with brace wear is certainly an issue, particularly for boys [6], and poor compliance has been associated with poor results [4, 7]. Furthermore, most children will have to wear the brace for an average of 2.9 years [8] but maybe for as long as 6 to 8 years, depending upon the patient's age at the start of bracing.

**Figure 1:**
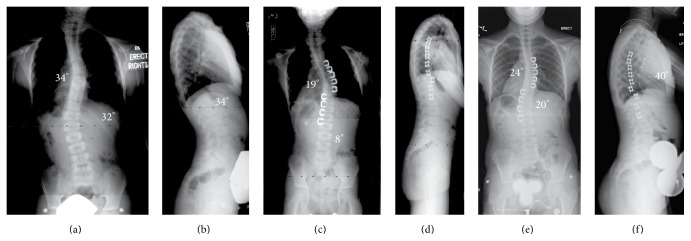
(a and b) This is an 8-year-old girl who presented with a double major curve (right thoracic: 34°, left thoracolumbar: 32°). The lateral X-ray shows a normal sagittal contour, with kyphosis from T5 to T12 of 34°. (c and d) Following VBS, the first erect PA and lateral films show the staples to be intact. There is excellent correction of both curves, with the thoracic curve correcting to 19° and the lumbar to 8°. (e and f) Films from 2-1/2 years postoperatively show successful maintenance of the thoracic curve (now measuring 24°) and the lumbar curve (now measuring 20°). The lateral film from 2 years postoperatively shows a 40° kyphosis from T5 to T12.

**Figure 2:**
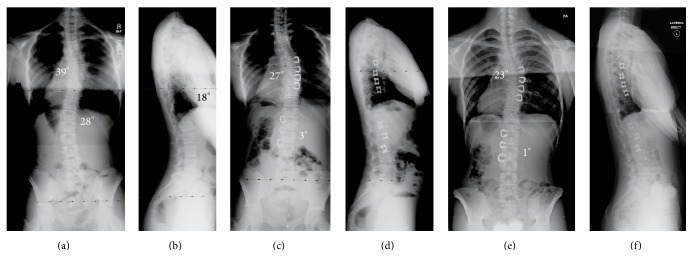
(a and b) This is an 11-year-old girl who presents with a 39° right thoracic curve and a 28° left lumbar curve. The lateral film shows 18° of kyphosis (normal sagittal contour) from T5 to T12. (c and d) Following VBS, the thoracic curve corrected to 27° and the lumbar curve to 3°. (e and f) Now at age 14, 2-1/2 years post-op, the patient is now Risser 4. The thoracic curve measures 23° and the lumbar 1°. The lateral film shows 21° of kyphosis from T5 to T12.

Vertebral body stapling (VBS) has been presented as an alternative treatment strategy for immature patients with IS (Figures 1 and 2). Preliminary results of VBS cohorts have been published [9, 10]. This current study reports a retrospective comparison study of VBS versus bracing alone for patients with moderate (25 to 44°) idiopathic scoliosis using identical inclusion criteria.

## 2. Materials and Methods

After obtaining IRB approval as well as informed consent for inclusion in research studies, we retrospectively reviewed our medical records from 2002 to 2007 and found a total of 83 patients who underwent VBS with proportional staples. The staple design was changed in 2002 to shorten the tines according to the disc height. Each staple had the correct proportional tine length as opposed to an earlier generation with a fixed tine length for all staples, which prevented the tines from being inserted close to the growth plate as was possible with the proportional staples. This study includes only patients having had surgery with proportional staples.

We identified 49 consecutive patients with 57 curves who met our inclusion criteria of (1) diagnosis of idiopathic scoliosis; (2) age at least 8 years at time of surgery or bracing; (3) curve measuring 25 to 44° at first visit; (4) Risser sign of 0 or 1 at the time of surgery; and (5) minimum 2-year follow-up after surgery. Of the 49 patients identified, 42 (86%) patients were available for this review. Seven patients had follow-up of less than 2 years, and further follow-up could not be obtained. VBS was performed on the largest curve, thoracic or lumbar, and the second curve was stapled if ≥25° and if the apex crossed the midline.

Data collection included patient age at the time of index procedure, gender, surgery/start of bracing date, and complications, if any. For the VBS group, measurements were made on preoperative posteroanterior (PA), lateral, and supine bending films at first erect and 2 years or longer recent follow-up. At each visit, standing PA and lateral radiographs were obtained. On the PA views, the Cobb angle and Risser grade were obtained. In addition, all radiographs were evaluated for signs of staple loosening, breakage, or dislodgment. This would include radiographic changes such as haloing around the staple prongs or subtle radiographic changes such that the staples are not in the exact same location from X-ray to X-ray.

The bracing cohort consisted of a consecutive series of patients derived from the Gothenburg Scoliosis Database which contains information about all patients with scoliosis (*n* = 2655) seeking advice or treatment at the Department of Orthopaedics at Sahlgren University Hospital in Gothenburg, Sweden, between 1963 and 1994. This database, previously used for other studies on patients who had been treated with brace or surgery for AIS [2, 11], was chosen as the source for a control group due to the ability to obtain a large control group with complete follow-up data. Most importantly, since it was an active database, it could be queried for identical inclusion criteria and for success criteria. Patients meeting identical inclusion criteria as the VBS group (curves < 45°) who had undergone brace treatment were sought for comparison. From this database, 154 curves in 121 patients treated between 1965 and 1990 were identified who met our inclusion criteria as outlined above. Until 1974, patients were treated with a Milwaukee brace (cervicothoracolumbosacral orthosis, or CTLSO) and then after 1974 a Boston brace (thoracolumbosacral orthosis, or TLSO). Patients were braced for at least 12 months, and the brace was recommended to be worn 22 to 24 hours per day until skeletal maturity.

Children in the braced group were found to be significantly older at start of bracing than the VBS group at surgery, 12.8 years versus 10.5 years, respectively (*P* < 0.0001). In order to make comparisons reliable, a subgroup from the Gothenburg database was constructed as a matched group based on the following matching criteria: initial Cobb angle, age at start of treatment, and Risser sign. This resulted in 52 patients with 66 curves in the braced group for comparison, and the age at the start of bracing was now not statistically significantly different between the two groups.

Patients with double curves, where one of the curves was included but the other one was too large for inclusion (i.e., >45°), were excluded from both the VBS and bracing groups.

A study by Katz and colleagues [12] examined the natural history and effectiveness of brace treatment for curves <45°. In this study, there seemed to be a significant division of results above or below 35°; therefore, 35° was chosen as the variable for curve severity subanalysis in our study of thoracic curves treated with VBS.

Each curve (thoracic or lumbar) was analyzed separately and grouped according to location of the curves, either thoracic (vertex down to T11) or thoracolumbar/lumbar (vertex from T11-T12 downward). “Improvement” was defined as improvement in the pretreatment Cobb angle of >10° measured on the final follow-up radiograph. “No change” was defined as a +10 to −10° change (both values inclusive) in the pretreatment Cobb angle. “Progression” was defined as worsening of the curve >10°. “Success” was defined as either “improvement” or “no change” on the final follow-up Cobb angle. Ten degrees was chosen so as to be clearly outside the range of possible measurement error due to radiographs being obtained and measured at different locations. In addition, based on a previous study comparing various types of braces [13], 10° was chosen to represent a clinically significant change in curve size.

If a curve progressed to posterior spinal fusion (PSF), this was considered a “failure” of treatment regardless of curve type. For example, if a patient had a double major curve pattern and then progressed to fusion, even if the lumbar curve had not progressed >10°, it was still considered a “failure.” This allows for the most conservative measurements for success of treatment and remains consistent with previous articles on bracing for scoliosis [1, 12, 14].

Statistical analysis was performed using the Statistical Analysis Systems (SAS) version 9.2 (SAS Institute, Cary, NC). GEE (Generalized Estimating Equation) was used to assess the occurrence of failure with regard to Cobb angle at the time of final measurement. The Wilcoxon two-sample test was used to assess demographic differences among the two groups. A *P* value of ≤0.05 was considered statistically significant.

### 2.1. Surgical Technique: VBS

While under general anesthesia, the patient is positioned in the lateral decubitus position with the convex side of the curve facing up. An axillary roll is used underneath the concave side above the curve so as to allow the curve to correct slightly. All vertebral bodies in the Cobb angle of the curve are instrumented. Single-lung ventilation and carbon dioxide (CO_2_) insufflation are used for better visualization. At this point, vertebral bodies are identified using biplanar fluoroscopy. The thoracoscope is placed through the anterior portal. Intercostal portals for staple insertion are placed close to the posterior axillary line. After incision, nasal speculum dilators are used to enlarge the portals. Under fluoroscopic control, a trial is used at every level to gauge the size of the staple. Optimal placement requires that the tines of the staple are close to the vertebral endplate. In the sagittal profile, the staple is placed anterior to the rib head. In patients with hypokyphosis, more anterior position is desired. In the lumbar spine, the staple is placed in the posterior half of the vertebral body. The tines of the trial are used to make pilot holes. The trial device can be used to push on the apex of the curve, thus improving the correction. Care is taken to protect the segmental vessels. After the holes are made, a staple of appropriate size (range, 3–8 mm wide in a 2- or 4-pronged design) is selected and its tines straightened using a distracter. Staples are placed in ice to keep the tines open. Then the trial is removed and the staple is quickly inserted using a specially made insertion device. Tines of the staple are matched with the pilot holes. Optimal position of the staple is reconfirmed fluoroscopically, and the staple is impacted into the vertebral body. After removal of the inserter, if the staple is not flush with the disc, an impactor is used to further drive the staple into the vertebral body. It is important to obtain optimal correction of the spine before impacting the trial or the inserter. If the spine is not straight, then an additional inserter can be placed in the previously inserted staple adjacent to the staple to be inserted and used to push on the spine (translation) to obtain curve correction.

In the lumbar spine, we have used a direct lateral, retroperitoneal approach with a minimal open incision [15]. Staples are placed at 3 to 4 levels. During the approach, the psoas is usually retracted posteriorly. As an alternative, one can gently separate the psoas muscle longitudinally directly over the posterior half of the disc under electromyographic control [15].

At the end of the procedure, position of the staples is reconfirmed using fluoroscopy. A chest tube drain is inserted under thoracoscopic guidance through the anterior portal.

In these patients, the chest tube was usually removed on the first postoperative day. Initially, a noncorrecting soft corset brace was prescribed for lumbar curves for 6 weeks to help with stabilization of the staples by decreasing the patients' motion. Activity restrictions were lifted after 6 weeks. Patients were observed radiographically every 3 to 4 months until skeletal maturity and then every 6 months thereafter.

## 3. Results

Demographics and radiographic appearance of the spinal deformity were compared in the two cohorts (Table 1). There were no significant differences between the two groups in terms of age and curve size at start of treatment. In the VBS group, of the 49 patients who met the inclusion criteria, 42 (86%) were located and data updated specifically for this study. The mean age at the time of surgery was 10.6 years (range, 8–14 years). The mean preoperative curve size was 31.5° (32° thoracic and 31° lumbar). Average follow-up was 40.8 months. There were 57 curves in 42 patients, with 15 patients having both thoracic and lumbar curves. For the bracing group, there were 66 curves in 52 patients, with 12 patients having both thoracic and lumbar curves and 2 patients having a double thoracic curve. The mean age at the start of treatment was 11.1 years. The curve size before start of bracing measured a mean of 31.3°. Average follow-up was 105 months (±37.8 months) after end of bracing; that is, at maturity.

For thoracic curves measuring between 25 and 34°, VBS had a success rate of 81% versus 61% for bracing (*P* = 0.16, Table 2). In thoracic curves measuring 35 to 44°, VBS had a very poor success rate of 18% versus 50% for bracing (*P* = 0.19). For lumbar curves, success rates were not different; for curves measuring from 25 to 34°, the success rate of VBS versus bracing was 80% versus 81%, respectively (*P* = 1.0), and for lumbar curves from 35 to 44°, the success rate was 60% versus 0%, respectively (*P* = 0.43). In the latter group, however, the numbers were low; only 5 patients underwent stapling, and 2 were braced.

We also examined the number of female versus male patients and determined that the small number of male patients (5 for VBS and 6 for the brace group) would not significantly alter our conclusions.

Complications were divided into 3 categories: major, minor, and insignificant [16]. There were no major complications in this series of patients. Minor complications in the VBS group consisted of 1 new postoperative foot dystonia that resolved within 3 months. One patient had 1 lumbar staple removed secondary to “backing out,” but it had not completely dislodged. Three broken staples were noticed, none of which required removal. All patients who started bracing were able to complete the treatment except for two who could not tolerate the bracing regimen due to psychosocial factors. The curves of both these patients progressed before maturity, and they were therefore surgically treated.

## 4. Discussion

Treatment options for moderate IS have long been limited to bracing and observation. The former commits the child either to 16–23 hours per day of brace wear or brace wear during the night. Either way, bracing is hot and uncomfortable, which is a particular concern in warmer climates and during summer months everywhere. Brace treatment typically lasts for 3 to 5 years but could be much longer for a patient who starts wearing a brace, say, at age 8. Serious self-image issues often accompany brace wear but have yet to be fully described in an objective manner. It has been shown that previously braced patients in their adulthood felt that their body asymmetry was worse than nontreated scoliosis patients, despite similar curve sizes, possibly an effect of long-term bracing before maturity [17, 18]. Furthermore, compliance with prescribed wearing schedules is also a factor that decreases the efficacy of brace treatment [7] (Karol LA et al.: “The effect of compliance monitoring on brace use and success in patients with AIS.” Paper presented at the POSNA Annual Meeting 2013, Toronto, Canada). Observation involves periodic radiographs and the anxiety associated with the possibility of progression.

The results of this study suggest that patients with some types of high-risk moderate scoliosis of 25 to 44° can be treated successfully with VBS. The best results were seen in thoracic curves measuring 25 to 34°, where VBS had a success rate of 81%, and in lumbar curves measuring 25 to 34°, where 80% of the patients were successfully treated with only few complications.

Treatment of larger thoracic curves measuring 35 to 44° at the start of treatment was not successful in any of the groups (18% for VBS versus 50% of the braced group). In the lumbar curves measuring 35 to 44°, we do not think that any conclusions can be drawn because the numbers of curves and patients were not large enough.

Results of bracing for idiopathic scoliosis are very dependent on the patient's age at discovery and at initiation of treatment. A recent review by Katz and colleagues [19] of 100 patients suggests that bracing over 12 hours per day was effective in 82%. All of the patients in Katz's study were over 10 years of age, and 10 of them were at Risser 2. Katz and colleagues' cohort was much more mature than the patients undergoing VBS in this current series (average age of 10.6 years). The recently published report by Weinstein and colleagues [4] included more mature patients, all of whom were older than 10 years of age, with a Risser degree of 0, 1, or 2. In contrast, Charles and colleagues [5] reported on a group of patients with juvenile idiopathic scoliosis who were treated with bracing. They found that for curves >30°, 100% of the patients required a fusion, and for those with curves 21–30°, 75% progressed to fusion despite bracing.

The negative aspects of brace wear are also often overlooked. Misterska and colleagues [20] have reported increased stress levels in patients and parents when bracing is utilized. Katz and colleagues [19] reported that the best results occurred when the patients wore the brace in the daytime and after school, a time when middle school students are often least likely to want to wear a brace. One of the indications for stapling thoracic curves measuring <35° or lumbar curves measuring 25 to 44° is when the child is distraught at having to wear the brace at school.

For this review, thoracic and lumbar curves were analyzed separately because each type of curve responds differently to both VBS and bracing. There were not enough patients to subanalyze the data by curve pattern (thoracic versus thoracolumbar/lumbar versus double major). Betz and colleagues [21] have demonstrated the feasibility, safety, and utility of the Nitinol staples in AIS. In another study, Betz and colleagues [9] reported results in 39 patients, in whom 87% of curves demonstrated coronal stability at a minimum 1-year follow-up. Betz and colleagues [10] have also reported a minimum 2-year follow-up for patients showing coronal stability of approximately 78% in all lumbar curves and thoracic curves <35°. Poor results were seen in thoracic curves >35° consistently in all the reviews.

This current paper shows similar results of failure with thoracic curves >35° with longer follow-up and more patients. This failure in thoracic curves >35° has led the senior authors to change strategies for VBS as a treatment option for thoracic curves >35°. One fusionless option to consider would be to use staples along with some kind of posterior growing system. The downside of this approach is the necessity to lengthen the posterior construct every 6 months. Maybe in the future, with noninvasive posterior lengthening systems, this may be a viable option. However, the authors' current strategy is to perform anterior vertebral body tethering alone for thoracic curves >35°.

We also now further understand the importance of maximizing correction on the OR table in that our best results (>80%) were seen when the first erect radiograph showed the curve reduced to <20° [10]. We now use intraoperative techniques to maximize correction (coronal and axial) of each curve segment before stapling each disc space. An example of this effort is maintaining sustained apical translation correction with the staple holder (inserter) as the more proximal levels are stapled. Because of the trend we have seen in obtaining correction, we now offer patients with significant growth remaining (Sanders digital score [22] <5) VBS as a corrective option and not just a means to hold the curve as an alternative to bracing. In addition, for a curve that does not measure <20° on the first post-op erect film, the patient should wear a nighttime corrective brace until the curve measures <20° or skeletal maturity is reached [10].

In evaluating the results of this study, both strengths and limitations must be taken into account. Two groups with identical inclusion criteria that were consecutive, had at least two years of follow-up, and were evaluated using the same methods constitute an ideal setting for a comparative study. A limitation of the study is that the average age at start of treatment of the patients in both groups was statistically different (10.6 years for VBS and 12.8 years for bracing; *P* < 0.001) when the groups were first compared. The statistician modified the braced patients included in the analysis in order to obtain a match in the average age of the patients. All patients were Risser 0 or 1 at start of treatment, which is only a rough estimation of skeletal maturity and proxy for the potential for scoliosis progression. Another limitation was that the control group was a historical group, that is, previously treated. However, being the only known way of achieving a large control group that had been meticulously followed and documented through the whole treatment period and with a sufficient follow-up time, this drawback had to be accepted. The VBS group was also limited in that the follow-up was only for 2 years, and the group was not followed to skeletal maturity. Minor changes in the results would be expected and will be the subject of a future study. Furthermore, we did not have hand X-rays for bone age or Sanders digital score. This would have been a much better comparison than age and Risser score but was not in use when treatment of these groups started. The coauthors currently use Sanders digital score <5 as an indication for recommending stapling.

For future study, genetic testing (when available) may also help with patient selection in further refining the subset of patients whose curves will definitely progress. Furthermore, genetic testing such as the Scoliscore may identify patients with curves that are likely to be refractory to bracing [23, 24]. Unfortunately, the positive predictive value of the Scoliscore has yet to be proven; therefore, this clinically available test is not yet helpful for predicting VBS as a desirable treatment for a genetically diagnosed high-risk curve.

Based on this review, the authors performing VBS currently use the following indications for recommending stapling to patients: (1) age <13 years in girls and <15 years in boys; (2) Sanders digital score <5; (3) thoracic curves ≤35° and lumbar coronal curves ≤45°; and (4) sagittal thoracic curve <40°. If the thoracic curve measures 35 to 45° and/or a thoracic curve measure <35° but does not bend below 20°, then vertebral body tethering is considered [25–28]. Also, if the curve on first erect film does not measure <20° after VBS, the patient should wear a corrective nighttime brace until it does.

## 5. Conclusion

In this comparison of two cohorts of patients with high-risk (Risser 0-1) moderate idiopathic scoliosis (measuring 25 to 44°), the results of treatment of smaller thoracic curves (25 to 34°) show VBS to have a better result by clinical trend but not statistically different results versus bracing (81% versus 61%, resp., *P* = 0.16). For lumbar curves measuring 25 to 34°, results appear to be similar for both VBS and bracing. These results suggest that VBS could be used as an alternative or adjunct to bracing for patients with these curve ranges who are struggling with the ramifications of brace wear. For thoracic curves measuring 35 to 44°, the number of patients in this group was too small for statistical evaluation.

## Figures and Tables

**Table 1:** Demographics and distribution of curves among included patients.

Criterion	Vertebral body stapling (*N* = 42)	Bracing (*N* = 52)	*P* values
Avg. age at start of treatment (years)	10.6 (8–14)	11.1 (8.4–11.5)	0.07

Avg. curve size at start of treatment (°)	31.5 (25–44)	31.3 (25–44)	0.82

Time of brace treatment (months)	—	40.8 (13–103)	—

Avg. follow-up after surgery/maturity (mos)	40.8 (23–75)	105 (46–185)	<0.0001

Number of females	52	60	
Number of males	5	6	

Thoracic curves 25–34°	26 (3M)^*∗*^	36 (2M)	

Thoracic curves 35–44°	11 (2M)	12 (3M)	

Lumbar curves 25–34°	15	16 (1M)	

Lumbar curves 35–44°	5	2	

^*∗*^M = Males.

**Table 2:** Results of treatment of patients with AIS treated with either VBS (57 curves) or bracing (66 curves).

	No change/improvement (%)	Progression (%)	*P* value
Thoracic curves 25–34°			
VBS (*N* = 26)	81	19	0.16
Bracing (*N* = 36)	61	39	
Thoracic curves 35–44°			
VBS (*N* = 11)	18	82	0.19
Bracing (*N* = 12)	50	50	
Lumbar curves 25–34°			
VBS (*N* = 15)	80	20	1.0
Bracing (*N* = 16)	81	19	
Lumbar curves 35–44°			
VBS (*N* = 5)	60	40	0.43
Bracing (*N* = 2)	0	100	
